# Ethics of assertive care in mental health: A gradual concept

**DOI:** 10.3389/fpsyt.2023.1083176

**Published:** 2023-02-23

**Authors:** Axel Liégeois

**Affiliations:** ^1^Faculty of Theology and Religious Studies, KU Leuven (Catholic University of Leuven), Leuven, Belgium; ^2^Organization Brothers of Charity, Gent, Belgium

**Keywords:** ethics, assertive care, decision-making capacity, responsibility, informed consent, coercion

## Abstract

Mental health professionals have a contradictory social mission: respecting the autonomy of persons with mental illness while at the same time providing them with unsolicited or assertive care when necessary. The aim of this contribution is therefore to reflect on the ethical question of how care professionals can provide assertive care in an ethically responsible manner. To answer this question, we take a relational view of human beings, draw on the Ethics Committee for Mental Health Care of the Organization Brothers, and invoke a case to shape the ethical reflection. In a relational view, assertive care starts by building a relationship of trust between the care partners: care users, next of kin and care professionals. We can distinguish different forms of assertive care based on the degree of decision-making capacity and the responsibility of the care users. The first two degrees of assertive care occur when care users are still fully capable of making decisions about care and of taking own responsibility: the care professionals [1] make themselves available for possible care, or [2] inform about possible care in the most objective way possible. When care users are partially capable of decision-making, the care partners share responsibility in six possible degrees of assertive care: the professionals [3] advise on possible care, [4] negotiate good care, [5] attract into assertive care, [6] persuade to assertive care, or exert [7] external or [8] internal pressure. If care users are completely incapable of decision-making in care, the care professionals and next of kin take on vicarious responsibility in two degrees of assertive care: the professionals [9] take over the care, or [10] carry out coercion. Which degree of assertive care is most appropriate must be considered in each situation. Criteria for determining the appropriateness of assertive care are the degree of decision-making capacity of the care users and the degree of the threat and seriousness of harm. As the threat of serious harm increases and the care users' decision-making capacity decreases, forms of assertive care with a more freedom-restricting character are ethically justifiable.

## Introduction

Assertive care is a relatively new form of care, first developed in the US in the late 1970s. The term refers to the Assertive Community Treatment, an intensive and highly integrated form of community-based mental health care (1). This care focuses on persons with severe mental illness in their home situation. Assertive means that care professionals vigorously and confidently determine which care is appropriate and necessary. They intervene by providing care to persons who are not seeking or are even avoiding care. This care is intended to increase their quality of life and to prevent them from causing nuisance in the community.

We believe that this assertive care does not only occur in an outpatient or home situation with persons with mental illness. Care professionals also undertake assertive care with care users in the many forms of residential care and in all sectors of care. This occurs not only in mental health care, but also in care for persons with intellectual disabilities and elderly persons with high dependency. In all these settings, there are persons who need care but do not explicitly seek it or avoid it.

Moreover, assertive care is a curious term. An ethical question is whether there is not always some form of assertion or interference in care. Assertiveness can be considered as a far-reaching form of commitment in the care relationship. Interference is always present to a lesser or greater extent in any care relationship because care professionals and care users are involved with one another. This relationship shows an ethical tension between a fundamental symmetry of equivalence and reciprocity and an actual asymmetry of unequal vulnerability, unequal dependence and unequal power (2). Viewed from this tension, care professionals who have more power because of their position, can be seen as always interfering in the care users' life. Even if care professionals do not sense this asymmetry and interference, care users will undoubtedly be aware of it. The asymmetry of the relationship inevitably leads to an experience of interference.

Assertive care is a challenge for mental health professionals. In society's current view of mental health care, persons with mental illness are seen as autonomous individuals with the right to make their own choices and as free citizens with the right to participate in community life. In this way, society gives mental health professionals a contradictory mission: on the one hand, to respect the freedom and autonomy of persons with mental illness by asking them informed consent for any care intervention, and on the other hand, to provide them with unsolicited or assertive care when necessary. The aim of this contribution is therefore to reflect on the ethical question of how mental health care professionals can provide assertive care in an ethically responsible manner.

## Methodology

### Relational view

To answer this ethical question, we start from a relational view of human beings. This is a first element of our ethical methodology. It is inherent to ethics to reflect critically on a human practice based on a fundamental vision of the human being. We opt for a relational view, in line with care ethics and personalism (3, 4). This is a well-considered option without denying that other approaches are possible. The dominant vision in Western society and mental health care is an individualistic one in which people primarily make their own choices and participate in social life according to their individual choices. A relational view of the human being, on the other hand, sees persons not only as autonomous individuals but also as relational beings. Not only their autonomy, but equally their connectedness with each other and with their environment is part of the essence of being human (5). Of course, people strive for autonomy in their lives. But human persons are equally connected to each other and to their environment through their mutual relationships. It follows from this actual and mutual connection that they are involved with each other.

Commitment is a fundamental attitude in which persons feel and realize that they belong and are connected. This attitude is not without implication. It leads to a sense of duty to bear the consequences of that connectedness. An important consequence is precisely that persons bear responsibility for each other. This means that they are called upon by the others' existence and humanity to respect these others, to provide necessary care and not to harm them. Being responsible for the others, however, does not lift the own responsibility of these others. It is a responsibility to enhance the other persons' responsibility (2). In a relational view of human beings, this is the foundation of assertive care.

### Ethical opinion

A second element of the methodology of this ethical reflection is that we rely on the opinion of the Ethics Committee for Mental Health Care of the Organization Brothers of Charity in Flanders, the Dutch-speaking part of Belgium (6). Although this Ethical Committee has only local authority, it has expert knowledge in the specific field of mental health care and its advice might have a broader relevance. The Ethics Committee consists of some 25 experienced care professionals and representatives of care users and family associations. We chair this Ethics Committee, play a coordinating role, conduct the literature review and edit the text of the opinion.

The Ethics Committee adopts a methodological approach that combines ethical discussion with literature review: participants' moral intuitions and practices are mutually confronted with insights from a number of scientific publications. We selected and reviewed the ethical literature on assertive care (7–16). In the first instance, committee members share and list their moral intuitions and practices. In the second step, they clarify their intuitions and practices, and critically evaluate them by comparing and contrasting them with each other and with insights from the literature. We then prepare a draft opinion, based on the ethical discussion and the literature review. In a fourth step, the members discuss that draft opinion and make changes. In the fifth step, the committee presents the new draft opinion to care professionals working in the field and care users and family associations, and integrates their comments and observations. Finally, the members discuss the draft opinion and make changes until they can reach a consensus. The entire process takes place in an open and free forum, allowing each participant to express their mind without any pressure based on authority or position.

This ethical opinion has a number of limitations. First, the opinion does not present a psychiatric view of assertive care, but limits itself to its ethical aspects, which is precisely the purpose of the opinion and this contribution. Second, there is little specific ethical literature on assertive care in contrast to psychiatric literature. Finally, the ethical discussion in the Ethics Committee is influenced by the culture of care and the share of residential care in the Organization of the Brothers of Charity. This was remedied by including care professionals from outpatient and home care on the Ethics Committee and by submitting the draft opinion to external care professionals and organizations.

### Case

Finally, we invoke a case in the methodology to shape the ethical reflection on assertive care. This case is not real but fictional. The case consists of elements of real cases that can no longer be identified. The purpose of the case is to highlight certain issues and illustrate ethical reflection, not to make a clinical case study.

Albert, a 48-year-old man, is referred by the social housing company to an assertive care team. The trigger is a severely neglected home, communication problems and confused contact. He had been admitted to a psychiatric hospital years ago because of psychosis. He was successfully treated but further psychiatric follow-up in the home context remained necessary. Albert, however, refuses this. He has a delusion in which he is convinced that all humans are programmed by aliens to destroy the world. He sees himself as an exception and claims to have special powers. He chooses to isolate himself from other humans and to live in harmony with nature. Therefore, utilities in his house are unnecessary and pigeons and vermin are allowed to nest in his house. In his communication, he is chaotic and associative. It is very difficult to instill a sense of reality in him.

Currently, there are pests in Albert's house again. Moreover, the technical inspection of the boiler is constantly delayed because he refuses access to his home. Because of the ongoing housing problems and despite repeated mediation attempts, the social housing company wants to take further legal action toward eviction. Therefore, the company is knocking on the assertive care team's door.

The ethical question is what form of assertive care is ethically responsible. What is the responsibility of the care professionals and Albert's responsibility? What degree of assertive care can professionals offer Albert?

## Relationships and network

### Relationship of trust

It follows from a relational view on the human person that professionals begin assertive care with creating connection and commitment by building a relationship of trust with the care users, and to the extent possible also with the next of kin (6). They can gain this trust by deliberately focusing on them. They look for what is at stake for the care users and try to improve their situation together with them.

In the case, care professionals focus on Albert's story and his potential needs. Driven by an ethical attitude of responsiveness, they approach Albert from his life perspective, showing their commitment and creating connection. Albert's concern is not his living conditions, but perhaps something else that the care professionals need to figure out and that may be related to his leisure activities, such as his interest in nature and animals. What is important to Albert? What is at stake for him? What are possible gains in life as he experiences it? The care professionals focus on Albert's questions first and try to find appropriate answers with him. In this way, according to Albert, the care professionals really take responsibility and his trust in them may grow. Only then can the care professionals ask him to also pay attention to issues that are important to them, such as his responsibility for his living conditions. The most important is building mutual trust and a dialogue about what is at stake for him.

### Next of kin

Since care users may be connected to people in their social network, it is important to include the latter in assertive care as well. Some of these stakeholders are “next of kin” (2). This means they have a relationship of trust and represent the interests of the care users. Next of kin are broader than relatives, and conversely, not all relatives are next of kin. They can play a particularly significant role in assertive care, and it is desirable that care professionals involve them as much as possible in the dialogue on that assertive care. This is only possible if care users agree or at least do not oppose it. In the remainder of our account, we do not mention the next of kin in assertive care every time. We assume that care professionals involve them in assertive care to the extent that the care users agree.

Anyway, in Albert's case, there are no next of kin. He lives alone and wants to isolate himself from others. Possibly care professionals could help search with Albert to make friends and re-establish past family ties, but this will not be easy because he does not want it himself for now.

## Gradations in decision-making capacity

Our guiding view is that human persons are connected and involved with one another. The commitment calls on the care professionals to respect the care users, to provide appropriate care and not to harm. Taking a relational view, care professionals bear responsibility for the care users, without losing sight of personal responsibility. This means that the care professionals, the care users and the next of kin all assume and share responsibility. From this position of shared responsibility, it is justifiable that professionals provide assertive care.

We can distinguish different degrees of responsibility that depend on the extent to which the care users are able to assume responsibility. The possibility to do this depends on their decision-making capacity, the ability to make free and considered decisions (17). Decision-making capacity is not black and white, but a gradual concept. We can situate it on a continuous line from full capacity to take decisions, *via* partial capacity, through to full incapacity. Moreover, decision-making capacity is not general but specific, and not constant but variable. This means that care professionals always evaluate it for a defined life domain or for a certain decision at a certain moment in time.

With Albert, the question is to what extent he is competent to make decisions about his living conditions and take responsibility for them. In the limited space of this contribution, we cannot make the complicated process of assessing his decision-making capacity. Therefore, we propose to assume that, due to his mental illness, Albert has neither full capacity nor full incapacity for his decisions, but has partial capacity tending toward incapacity to take responsible decisions.

## Gradations in responsibility

Because of this gradual and specific concept of decision-making capacity and thus of the extent to which the care users can take responsibility for a certain decision, we can distinguish between different forms of responsibility (6). There is a gradation on a continuous line from personal, through shared, to vicarious responsibility.

On one side of the continuous line is the personal responsibility of the care users who are fully capable of taking a specific decision at a specific moment in time. Care professionals have the task of stimulating care users to take responsibility. The professionals' responsibility may not limit the care users' responsibility, but should support and promote it. The care users bear responsibility for their own life and to a certain extent also for the lives of others to whom they are connected.

On the other side of the continuous line, the vicarious responsibility of the care professionals is for the care users who are fully incapable of decision-making for a specific decision at a specific moment in time. In some situations, care professionals are required to take over the vicarious responsibility of the care users when required and for those life domains or decisions which are beyond them. Because the level of interference is high, this is a far-reaching form of assertive care.

Between personal and vicarious responsibility there is a gradation of shared responsibility among care users who are partially capable of decision-making at a given time and for a specific decision. A gradual concept of shared responsibility creates space for assertive care without removing or doubting one's personal responsibility.

## Gradations in assertive care or gradual care

**Figure 1:**
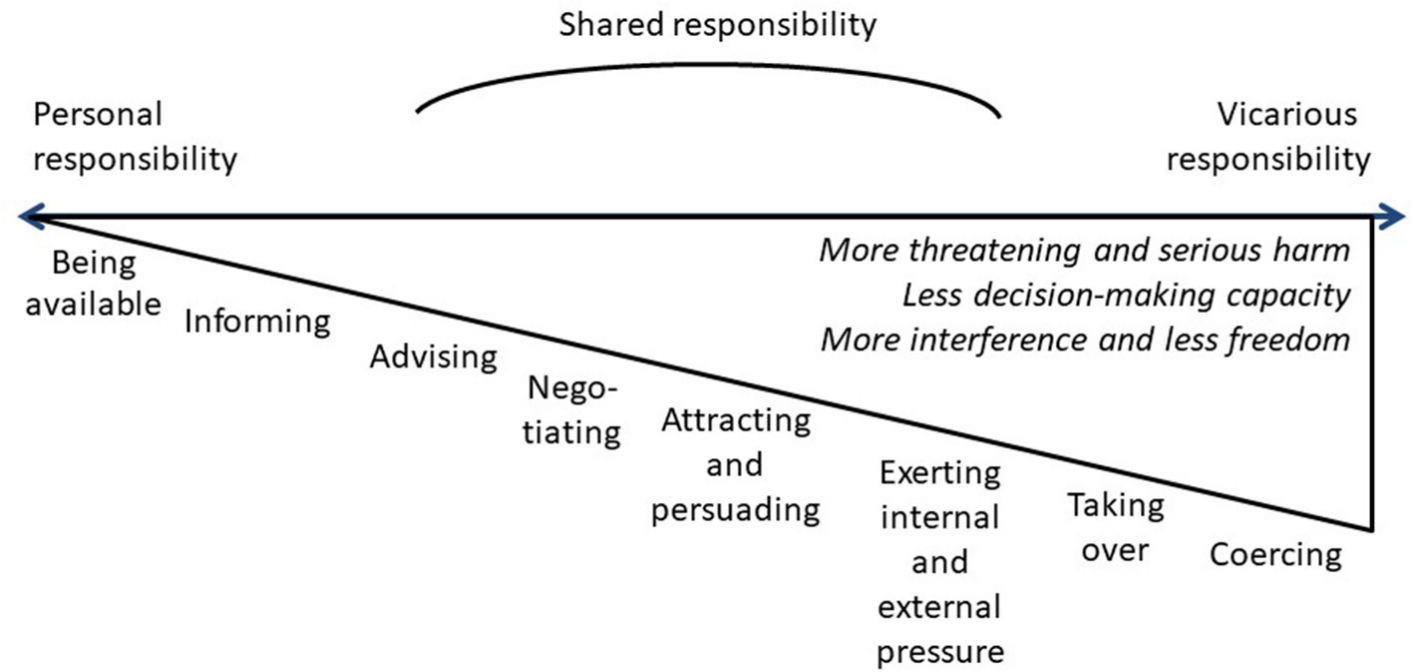
Assertive or gradual care.

The different degrees of shared responsibility lead to different degrees of assertive care (6). On the gradual line of responsibility, we distinguish various forms of care. We call this “gradual care” and therefore argue for a gradual concept of assertive care. Care professionals can choose the most appropriate form in each situation. In gradual care, based on the ethical discussion in the Ethics Committee and the literature review, we may discern a continuing line toward ever greater involvement: from being available and informing, through advising, negotiating, attracting and persuading, or exerting external or internal pressure, to taking over and coercing. All these forms of gradual care are valuable. Depending on the situation, care professionals can apply the most appropriate form of gradual care. We show the gradation in the forms of gradual care in Figure 1.

We now elaborate on this gradual view of decision-making capacity, responsibility and assertive care and apply it to Albert's case.

### Personal responsibility

If Albert were fully capable of making decisions, he could assume full responsibility. Then care professionals would not need to enter into a care relationship with him to support his responsibility. Here we can already distinguish two preparatory or preliminary forms of assertive care. However, they do not apply to Albert.

#### Being available

A first gradation in gradual care concerns availability. The care users can take personal responsibility and have not yet requested care. The care professionals let the care users make their own decisions and respect their autonomy. They make themselves available or willing to provide care, should that be necessary. This preparatory form of assertive care is not an issue with Albert.

#### Informing

Providing information is a second form of gradual care in the transition from personal to shared responsibility. The care users can fully assume responsibility and do not yet rely on care. They can ask for information or the care professionals may take initiative to give information. The care professionals inform the care users by providing information in the most objective way possible about the various possible actions with their related advantages and disadvantages. The care professionals do not try to influence the care users and do not give advice, but leave them completely free and encourage personal responsibility as much as possible. Informing as a preparatory form of assertive would no longer have any effect on Albert.

### Shared responsibility

If Albert has partial decision-making capacity for his living conditions, care professionals come to shared responsibility in dialogue with Albert and his next of kin, if any. Here we can distinguish different forms of assertive care.

#### Advising

A next gradation in gradual care is giving advice. Again, the care users can ask for advice or the care professionals can give advice on their own initiative. When it comes to advice, there is already shared responsibility. As with informing, care professionals give the care users information about the various choices and their pros and cons, but they now express their preference in the form of advice. They motivate this by emphasizing the advantages of a certain option and therefore the advantages for the care users. However, they leave the choice free without further attempts to influence. This first form of assertive care may have been applied to Albert by care professionals in the past, but advising him now would no longer have any effect.

#### Negotiating

Negotiation is a fourth form of gradual care that is located almost in the middle of the gradual line. Care professionals provide information about the different, possible choices, chart their pros and cons, and take stock of them. They do this without manipulating the information or presenting the choices differently. They formulate a proposal and give arguments for it. They listen to the emotions and feelings, thoughts and opinions of the care users. They try to meet the care users' wishes by adjusting the proposal and providing reasonable arguments. They strive for a consensus, with a balance that is as positive as possible for all parties involved. It would be wonderful if this negotiation with Albert could still succeed because there would be a balance between Albert's input and that of the care professionals. If the relationship of trust can grow and his decision-making capacity can increase, this form of gradual care could still be tried.

#### Attracting and persuading

Another degree of gradual care is attracting and persuading. As with advising and negotiating, the care professionals provide information and give their advice and arguments, but they now try to influence and motivate the care users more directly in the direction of their preferred option. They place even more emphasis on the benefits. They try to influence the care users, and this can be done both by attracting and persuading. In attracting, care professionals entice the care users across the line, by emphasizing the emotionally attractive elements and benefits of an option. In persuasion, they push the care users over the line, as it were, by working with reasonable arguments and advantages in favor of the proposed choice. However, in attracting and persuading, the emotional and the rational are mixed up together. It is also difficult to determine where the interference is greatest. However, care professionals do not manipulate the information, nor do they present the choices in a falsely positive light. These gradations of assertive care still have a chance to succeed with Albert. Care professionals must then be able to demonstrate attractive aspects of the changed living conditions. Or they must be able to convince him of the need for this change. If they succeed in this, they will continue to respect him strongly without exercising any real pressure.

#### Exerting external and internal pressure

The next form of gradual care is pressuring. Once again, the care professionals provide information as objectively as possible about the various choices with their pros and cons, and take stock of them. But now they go a step further by confronting the care users with the drawbacks of decisions other than those they propose. They do not manipulate the information and do not present the choices in an overly negative way. They do confront care users with the negative effects of other possibilities and use this as a lever to put pressure on them to select their own proposal. The interference increases and emphasis is placed on the disadvantages of a particular choice. The care professionals can emphasize the negative effects of decisions or measures taken by “external bodies,” such as people from the social network or societal actors: a homeowner or employer, a representative of a bank or insurance company, police or judicial authorities. Then they mediate to try and minimize the disadvantages. Based on their role as an “ally,” they look with the care users for ways to avoid the negative effects of external decisions or measures. If care professionals also exert pressure *via* the negative effects of decisions or restrictive measures that they are considering taking themselves, then they directly exert “internal pressure.” This makes the alliance with the care users more difficult. But even then they try to avoid any disadvantages. In a benevolent way, they can examine with the care users how best to avoid decisions or measures that might have negative effects.

Exerting external pressure is exactly what happens in Albert's case. The social housing company wants to take legal action toward eviction and therefore turns to the care professionals. The latter use “external pressure” by taking the negative effects of eviction as a leverage to change Albert's attitude and behavior toward his living conditions. There is no “internal pressure” in the case. It would be if the care professionals pointed out to him that the social housing company would evict him if they informed the company about his living conditions.

### Vicarious responsibility

When Albert becomes completely incapable to make decisions about his living conditions, care professionals take over responsibility vicariously. So far they have only consulted, but now they are moving to take action.

#### Taking over

A penultimate degree of gradual care is taking over responsibility. Until now the care professionals have only considered verbal interventions: being available, informing, advising, negotiating, attracting, persuading and exerting external or internal pressure. Now they are going to act. They take over responsibility from the care users and motivate their decision or measure. Contrary to coercion, the care users do not oppose this. It is not their wish or will. However, they accept or undergo the intervention without explicitly opposing it. This is a risky intervention in terms of interference because care professionals may have the impression that the care users agree simply because there is no opposition. They are now moving from guidance about choices to steering and structuring the life of the care users.

So far, this taking over has not happened in Albert's case. It would mean that the care professionals unilaterally decide that the house should be cleaned or the boiler checked by external professionals with whom they make an appointment. Specific to this taking over is that Albert would undergo these actions and not oppose them, even if he did not agree with them.

#### Coercing

The final form of gradual care is the most far-reaching, namely coercion. Care professionals take a decision or coercive measure and motivate it. They carry out the decision even if it explicitly goes against the will of the care users and even if they oppose it with words or actions. This is where interference is greatest and freedom is most restricted. Because coercion is so far-reaching, it should never become an automatic measure. Coercion must always be justified. We have elaborated on three conditions for the exercise of coercion (2, 18). The first condition concerns the degree of threat and seriousness of harm to the physical and/or mental health and/or integrity of the care users and/or other persons. The purpose of the coercive measure is to prevent or repair the harm. The second condition is considering the decision-making capacity of the care users to enter into dialogue or to control their own behavior. The purpose of the coercive measure is then to create a care situation in which the decision-making capacity is restored and promoted. The third condition is the proportionality of the coercion and its consequences in relation to the degree of threat and seriousness of harm and the degree of decision-making capacity. This implies that coercion can only be justified if there are no alternatives to avoid the threat and serious harm with less interference or restriction of freedom.

It would be debatable whether these criteria were met in Albert's case. How serious and threatening is the danger to his health and integrity and those of others? How limited is Albert's decision-making capacity to engage in dialogue? Is there a reasonable relationship between the harm caused to Albert by the coercion and the harm avoided by the coercion? Are there no alternatives that could remedy the problem of living conditions with less coercion? Perhaps there are alternatives after all, although this would have to be assessed in the very particular circumstances of a care user like Albert.

## Appropriate forms of assertive care

There is a gradation in these forms of gradual care or assertive care. Based on the relationship of trust, care professionals try to empower the care users to take responsibility for a certain choice. Of course, it is best to choose the form of gradual or assertive care with the most autonomy for the care users. But if the situation becomes more difficult, care professionals can opt for forms of gradual care with greater interference, and less personal and more vicarious responsibility.

The criteria for the appropriateness of gradual or assertive care are the extent to which the choice of a particular form corresponds to the degree of the care users' decision-making capacity, and the degree of threat and seriousness of harm that can be caused. These two criteria are derived from the conditions for coercion. This is quite obvious because the conditions for gradual or assertive care become most evident in its most extreme form, precisely in coercion (6).

The greater the vicarious responsibility in a form of gradual care, the greater the interference with and limitation of the freedom of the care users, up to and including the possibility of coercion. Care professionals can only justify this if they take the two criteria into account. The first is the degree of decision-making capacity. It is justifiable to choose forms of care with less personal responsibility and more shared or vicarious responsibility if the care users are less capable to make a responsible decision at a specific moment in time. The second criterion is the degree of threat and seriousness of harm. As the threat and seriousness of damage increase, it is justifiable to choose forms of care with less personal responsibility over shared to vicarious responsibility.

We can state that it is responsible for care professionals to choose forms of care with more interference as the care users become less capable of decision-making and the threat and seriousness of harm increase. Care professionals may be acting in an ethically irresponsible way by interfering too much in relation to the degree of decision-making capacity and damage, but it can also be irresponsible to interfere too little, overestimate the care users' capacity for personal responsibility and take insufficient steps to prevent threat and serious harm. After all, the evaluation of decision-making capacity must take into account the seriousness of the consequences, and therefore the threat of harm.

The choice of an appropriate form of gradual or assertive care must be carefully considered in each situation. It is important to apply this choice in a consistent manner. As far as possible, this choice is made in a dialogue between the partners in care: the care users if they are sufficiently decisive, the next of kin and the care professionals. They should also evaluate and review the choice, taking into account the evolution of the care users and their context.

What does this mean for Albert's case? This is difficult to assess because we would need much more information about his life history and life circumstances. We have argued that we can assess his decision-making capacity as partially capable, tending toward incapacity to make responsible decisions. We can consider the threat and seriousness of harm as decent, but also not absolutely dangerous. Indeed, there is no acute danger to the health or integrity of Albert or any other person, even though that danger may increase and it remains difficult to assess that danger at any time. Therefore, based on these premises, it seems that the external pressure from the care professionals and the housing company is justified, that taking over and exerting coercion would be excessive for the time being, and that be best that the care professionals can still do is to try to negotiate, attract and persuade.

## Conclusion

Assertive care is not an easy task: care professionals provide unsolicited care and this is not obvious if, at the same time, they want to fully respect the autonomy and wishes of care users. To justify this, we explicitly adopt a relational view of human beings. This view emphasizes connectedness and commitment, and thus responsibility for each other without taking over personal responsibility. This vision also prompts us to first build a trusting relationship as a basis for assertive care, and to involve the next of kin in this care.

Starting from this relationship of trust, care users, next of kin and care professionals come to assume responsibility. They can take their responsibility in relation to the degree of their decision-making capacity. According to this capacity, we can distinguish a continuous line between personal, shared and vicarious responsibility. Assuming responsibility takes many forms and gradations with increasing involvement: from being available and informing, through advising, negotiating, attracting and persuading, exerting external or internal pressure, to taking over and exerting coercion. Care professionals choose the appropriate form of assertive care, taking into account the degree of decision-making capacity and the degree of threat and seriousness of harm. The more threat of harm and the less decision-making capacity, the more it is justified to choose assertive care with more interference. However, assertive care remains an ethical challenge and an opportunity to grow in professionalism.

## Author contributions

The author confirms being the sole contributor of this work and has approved it for publication.
